# Origin and Characteristics of Internal Genes Affect Infectivity of the Novel Avian-Origin Influenza A (H7N9) Virus

**DOI:** 10.1371/journal.pone.0081136

**Published:** 2013-11-22

**Authors:** Yan Feng, Haiyan Mao, Changping Xu, Jianmin Jiang, Yin Chen, Juying Yan, Jian Gao, Zhen Li, Shichang Xia, Yiyu Lu

**Affiliations:** Key Lab. of emergency detection for Public Health of Zhejiang province, Zhejiang provincial Centre for Disease Control and Prevention, Hangzhou, Zhejiang province, People’s Republic of China; Thomas Jefferson University, United States of America

## Abstract

**Background:**

Human infection with a novel avian-origin influenza A (H7N9) virus occurred continuously in China during the first half of 2013, with high infectivity and pathogenicity to humans. In this study, we investigated the origin of internal genes of the novel H7N9 virus and analyzed the relationship between internal genes and infectivity of the virus.

**Methodology and Principal findings:**

We tested the environmental specimens using real-time RT-PCR assays and isolated five H9N2 viruses from specimens that were positive for both H7 and H9. Results of recombination and phylogeny analysis, performed based on the entire sequences of 221 influenza viruses, showed that one of the Zhejiang avian H9N2 isolates, A/environment/Zhejiang/16/2013, shared the highest identities on the internal genes with the novel H7N9 virus A/Anhui/1/2013, ranging from 98.98% to 100%. Zhejiang avian H9N2 isolates were all reassortant viruses, by acquiring NS gene from A/chicken/Dawang/1/2011-like viruses and other five internal genes from A/brambling/Beijing/16/2012-like viruses. Compared to A/Anhui/1/2013 (H7N9), the homology on the NS gene was 99.16% with A/chicken/Dawang/1/2011, whereas only 94.27-97.61% with A/bramnling/Beijing/16/2012-like viruses. Analysis on the relationship between internal genes and the infectivity of novel H7N9 viruses were performed by comparing amino acid sequences with the HPAI H5N1 viruses, the H9N2 and the earlier H7N9 avian influenza viruses. There were nine amino acids on the internal genes found to be possibly associated with the infectivity of the novel H7N9 viruses.

**Conclusions:**

These findings indicate that the internal genes, sharing the highest similarities with A/environment/Zhejiang/16/2013-like (H9N2) viruses, may affect the infectivity of the novel H7N9 viruses.

## Introduction

Human infection with a novel avian-origin influenza A (H7N9) virus, which is associated with severe respiratory symptoms and even deaths, was first reported in eastern China in April, 2013 [1,2]. There have been 135 diagnosed cases including 44 deaths as of Aug 14^th^, attracting great attention worldwide [3]. 

The novel H7N9 virus is a triple reassortant virus, in which the HA and NA genes originated from A/duck/Zhejiang/12/2011 (H7N3) and A/wild bird/Korea/A14/2011 (H7N9) respectively, whereas the internal genes are closely related to A/brambling/Beijing/16/2012-like viruses (H9N2), as previously described [1]. Most of the current researches have focused on the HA and NA genes since the Q226L mutation in the HA protein has been considered to change the binding capacity from avian species to human, and thus might increase the transmission ability in air [4-6]. However, various studies have also shown that the continuous reassortments occurred on the internal genes of avian influenza A virus played a key role in the direct interspecies transmission and triggering human infection [7,8]. This study, therefore, paid close attention to the origin and characteristics of the internal genes of the novel H7N9 virus.

Different subtypes of avian influenza viruses (AIVs) possess different virulence and infectivity. Except for domestic poultry and wild birds, some AIVs including subtype H5, H7 and H9 had been detected from humans [9]. The high pathogenic avian influenza (HPAI) H5N1 viruses can spread rapidly in and between poultry, resulting in hundreds of millions of domestic birds affected and killed [10]. Only in China, there are approximately 100,000 domestic birds infected with the H5N1AIVs every year, causing huge economic losses [11]. The HPAI H5N1 viruses can also be widely transmitted by poultry products, poultry movements and migration of wild birds. In total of 63 countries had reported to detect the HPAI H5N1 viruses from poultry or wild birds [12]. In addition, the HPAI H5N1 virus is a great threat to human as 637 human beings had been infected since 2003 [13]. 

In contrast to H5N1, subtype H9 AIVs were generally considered to be low pathogenicity viruses causing mild disease among domestic poultry and wild birds [14-16]. Human infections with H9N2 AIVs have occasionally been reported in southern China and Hong Kong, but the clinical symptoms of the patients were mild to moderate and no deaths have occurred [17-19]. Even low pathogenic, H9N2 AIVs, however, possess high infectivity in both poultry and human. Since the first subtype H9N2 AIV was isolated in 1966 [20], the H9N2 AIVs have been monitored from multiple avian species in various regions [21-26]. The previous serological surveys have also pointed out that the positive rates for anti-H9N2 antibody were high in both poultry and human beings. In Iran, 23% to 87% of poultry-related workers possessed antibody for H9 [9]. Chinese studies also reported that 12.8% of chickens and 5.1% of poultry-related workers in Guangzhou area were seropositive for H9N2 [27]. Even in healthy individual, the prevalence of anti-H9N2 antibodies was reported to be around 2% [19,28]. 

Infections with earlier H7N9 AIVs were rarely reported in China or sporadically happened in a couple of countries [29,30]. No H7N9 virus has been monitored and isolated from poultry in Zhejiang province before 2013, which indicated the low transmissibility of the earlier H7N9 AIVs. However, the novel H7N9 viruses represented high transmissibility as the HPAI H5N1 and the H9N2 AIVs possessed. Our concern is whether we can find any common features on the basis of the genome sequences among the HPAI H5N1 viruses, the H9N2 and earlier H7N9 AIVs, as well as the novel H7N9 viruses, which could be the possible reason for the high infectivity of the novel H7N9 virus. 

In this study, we monitored the environmental specimens collected from live poultry markets and isolated five avian H9N2 viruses from both H7 and H9 positive specimens. Meanwhile, the origin of the internal genes of the novel H7N9 virus was systematically illustrated by analyzing the entire sequences of 221 influenza viruses using RDP3 software. Phylogeny analysis and homological comparison were performed to determine the reassortment events. In addition, the specific amino acids possibly related to the high infectivity of the novel H7N9 viruses were analyzed by comparing the genome sequences with the HPAI H5N1 viruses, the H9N2 and the earlier H7N9 AIVs.

## Results

### Surveillance of AIVs from the environmental specimens

 After the outbreaks of human infection with the novel H7N9 virus in Zhejiang province, China in 2013, we tested the environmental specimens collected from live poultry markets using real-time RT-PCR. Within the 82 environmental specimens collected from six cities, 33 (40.24%) were found to be positive for H7, 15 (18.29%) positive for H5 and 39 (47.56%) positive for H9. There were 23 (28.05%) found to be positive for both H7 and H9. Of the specimens positive for both H7 and H9, five AIVs were isolated, including A/environment/Zhejiang/09/2013 (ZJ09), A/environment/Zhejiang/13/2013 (ZJ13), A/environment/Zhejiang/14/2013 (ZJ14), A/environment/Zhejiang/15/2013 (ZJ15) and A/environment/Zhejiang/16/2013 (ZJ16), in which two specimens were from Hangzhou city and three from Huzhou city. Ct values of the clinical specimens of the five isolates ranged from 10 to 14 for H9, and 17 to 35 for H7. After the virus isolates were obtained from allantoic liquids, the whole genome of the isolates were sequenced and aligned in Blast of NCBI. The results showed that the sequences of each gene shared the highest similarities with H9N2 AIVs strains, and thus the five Zhejiang isolates were identified as avian influenza A (H9N2) viruses. The details of five Zhejiang H9N2 AIVs are shown in Table 1.

**Table 1:** Details of the avian influenza A (H9N2) virus strains isolated from environment specimens in Zhejiang province, China in 2013.

				**rRT-PCR (Ct value)**			
**No.**	**Strain name**	**Source location**	**Specimens**	**H7**	**H9**		**GenBank accession No.**
1	A/environment/Zhejiang/09/2013	Hangzhou	Chicken manure	35	14		KF178662-KF178669
2	A/environment/Zhejiang/13/2013	Hangzhou	Swab of chicken cage	29	10		KF178670-KF178677
3	A/environment/Zhejiang/14/2013	Huzhou	Swab of chicken cage	17	10		KF178678-KF178685
4	A/environment/Zhejiang/15/2013	Huzhou	Sewage of poultry market	22	11		KF178686-KF178693
5	A/environment/Zhejiang/16/2013	Huzhou	Chicken manure	29	11		KF178694-KF178701

rRT-PCR is the abbreviation of real-time reverse-transcriptase polymerase chain reaction

H7and H9 means the influenza virus subtype H7 and H9.

GenBank accession numbers of each strain contain eight numbers for the HA, NA, PA, NP, M, NS, PB1 and PB2 genes.

A retrospective analysis on surveillance data for the AIVs in Zhejiang province between 2011 and 2012 was performed as well. In total 784 environmental specimens were tested by real-time RT-PCR assay to detect H5, H7 and H9 AIVs in 2011, in which 13 (1.66%) were positive for avian H5, 34 (4.34%) were positive for H9 and three (0.38%) were positive for both H5 and H9. No H7 positive specimens were detected. In 2012, 709 specimens were tested, with 26 (3.67%) positive for H5 and 47 (6.63%) positive for H9. The detection of H7 was not performed. 

### Origin of the internal genes of the novel H7N9 virus

**Figure 1:**
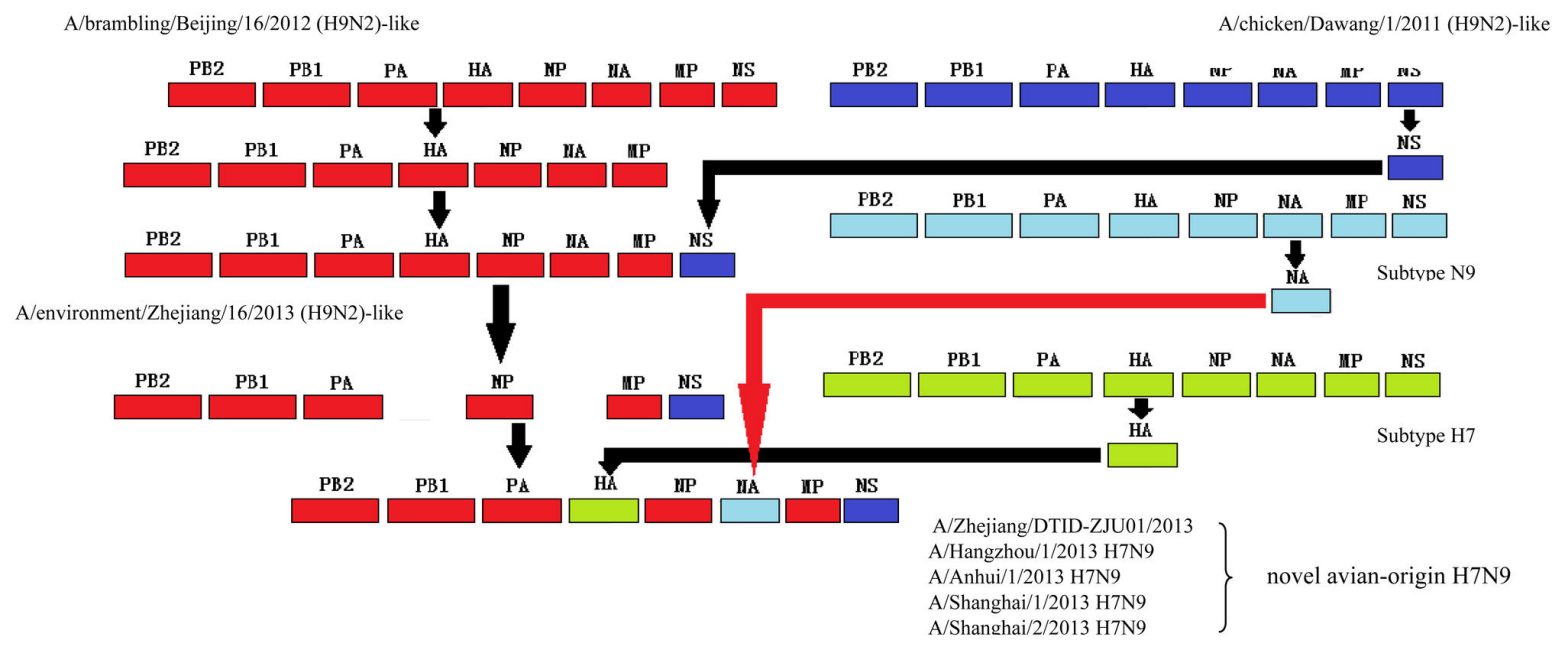
Evolutionary process of the novel H7N9 virus isolated in China in 2013. Reassortment events on the six internal genes of the novel H7N9 viruses were analyzed based on the entire sequences of 221 influenza viruses using the RDP, GENECONV and MaxChi suites within the RDP3 software. In order to enhance the credibility of the results, the highest P value was set as 0.01. Note: A/brambling/Beijing/16/2012 (H9N2) -like strains including A/chicken/Shangdong/01/2009, A/chicken/Jiangsu/Q3/2010, A/chicken/Zhejiang/611/2011, A/chicken/Shanghai/C1/2012, A/chicken/Zhejiang/329/2011 and A/chicken/China/AH-10-01/2010. A/environment/Zhejiang/16/2013 (H9N2) -like strains including A/environment/Zhejiang/9/2013, A/environment/Zhejiang/13/2013, A/environment/Zhejiang/14/2013, A/environment/Zhejiang/16/2013. A/chicken/Dawang/1/2011 (H9N2)-like strains including A/chicken/Shuanggou/1/2011 (H9N2) and A/pigeon/Xuzhou/1/2011. The eight genes of different strains were shown in different color.

The evolutionary process of the novel H7N9 virus analyzed by RDP3 software showed that the six internal genes of the novel H7N9 viruses shared the highest similarities with ZJ16 (H9N2)-like viruses (Figure 1). Significant reassortment signals were found on the internal genes of ZJ16 (H9N2)-like viruses, with acquiring NS gene from a H9N2 virus isolated from chicken in Jiangsu province in 2011, named A/chicken/Dawang/1/2011 (H9N2), and other five genes from A/brambling/Beijing/16/2012 (BJ16) (H9N2)-like strains. 

Comparisons on the nucleotide sequences amongst A/Anhui/1/2013 (AH1) (H7N9), ZJ16 (H9N2), BJ16-like viruses and A/chicken/Dawang/1/2011 are summarized in Table 2. The homologies on the HA and NA genes between AH1 (H7N9) and ZJ16 (H9N2) were 51.45% and 54.25% respectively, whereas the homologies on the six internal genes ranged from 98.98% on the MP gene to 100% on the NP gene, which were significantly higher than the homologies between AH1 and BJ16. Focusing on the NS gene, AH1 shared a 99.16% similarity with A/chicken/Dawang/1/2011 (H9N2), however, only 94.27~97.61% with BJ16 (H9N2)-like viruses.

**Table 2:** Homology comparisons on six internal genes between the novel H7N9 virus A/Anhui/1/2013 and six H9N2 AIVs.

	**Homologies on each gene (%)**							
**Strains**	**HA**	**NA**	**MP**	**NP**	**NS**	**PA**	**PB1**	**PB2**
A/environment/Zhejiang/16/2013	51.45	54.25	98.98	100	99.40	99.91	99.78	99.87
A/chicken/Dawang/1/2011	52.01	52.88	98.15	96.28	99.16	94.81	97.33	95.53
A/chicken/Jiangsu/Q3/2010	52.18	53.30	98.47	98.73	96.42	97.58	98.90	98.07
A/chicken/Zhejiang/611/2011	51.71	52.95	97.86	98.86	94.87	96.79	97.71	95.57
A/chicken/Zhejiang/607/2011	51.92	52.67	98.57	97.33	94.27	98.74	98.37	97.15
A/brambling/Beijing/16/2012	51.74	53.23	97.96	97.39	97.61	99.16	98.68	99.25

**Figure 2:**
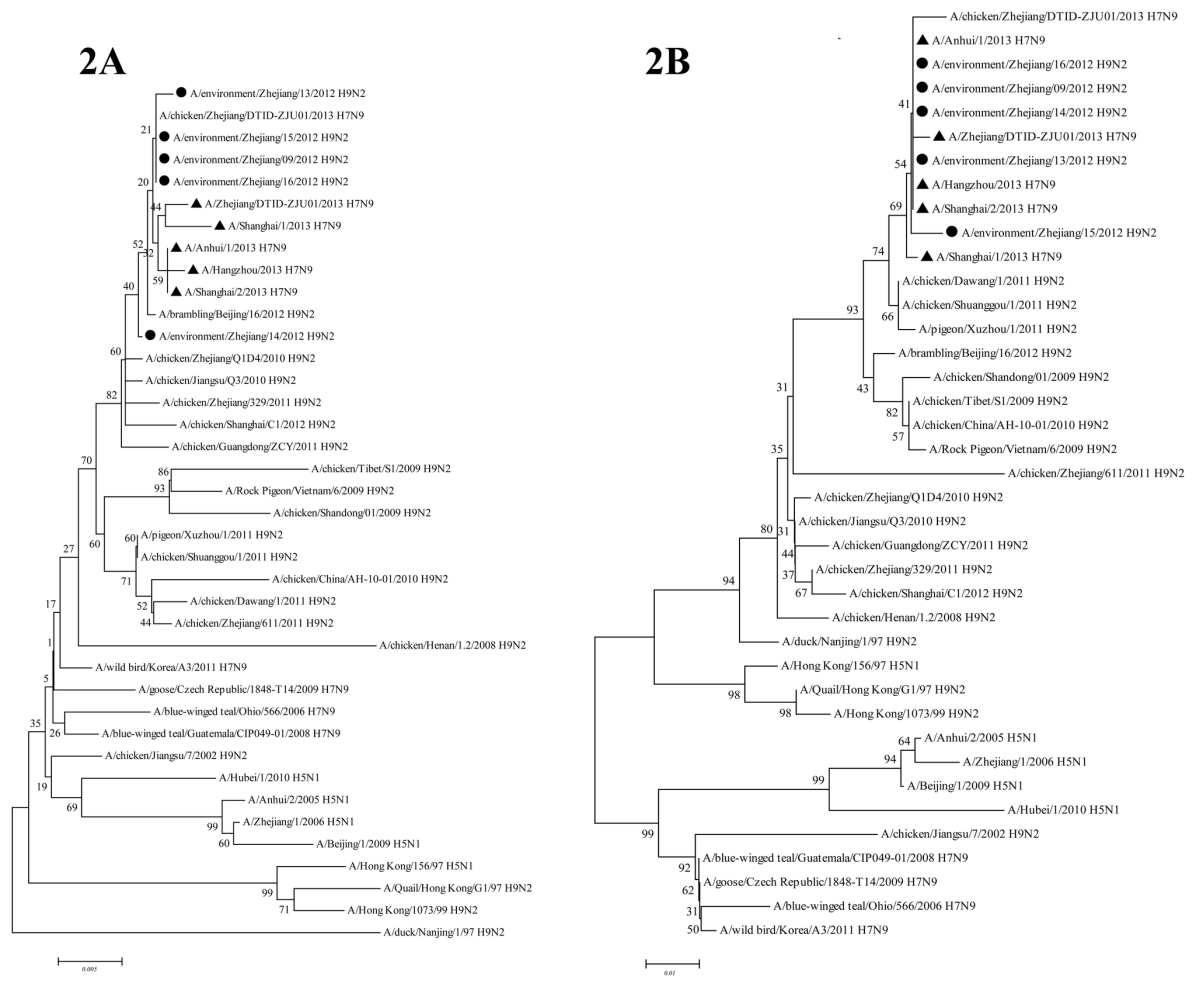
Phylogenetic trees for PB2 and NS genes of the novel H7N9 viruses. Figure 2A and 2B was constructed based on the amino acid sequences of PB2 and NS genes respectively. These trees were all estimated using the Neighbor-joining (NJ) method of MEGA software (Version 5.0) with the bootstrap analysis of 1000 replications. The novel H7N9 isolates are marked with a triangle and the H9N2 virus strains isolated from environment specimens in Zhejiang province in 2013 are marked with a circle.

Phylogenetic trees drawn on the basis of the amino acid sequences were used to confirm the reassortment events. The PB2 gene phylogenetic tree (Figure 2A) showed that five novel H7N9 strains were closest to Zhejiang H9N2 strains ZJ09, ZJ13, ZJ15 and ZJ16, but also located in the same branch with BJ16 (H9N2). The same results were obtained in MP, NP, PA and PB1 genes (results not shown). However, in the NS phylogenetic tree (Figure 2B), the closest strains to the novel H7N9 strains and five Zhejiang H9N2 isolates were A/chicken/Dawang/1/2011 (H9N2), A/chicken/Shuanggou/1/2011 (H9N2) and A/pigeon/Xuzhou/1/2011, three strains isolated from Jiangsu province in 2011.

### Amino acids associated with the high infectivity of the novel H7N9 virus

**Figure 3:**
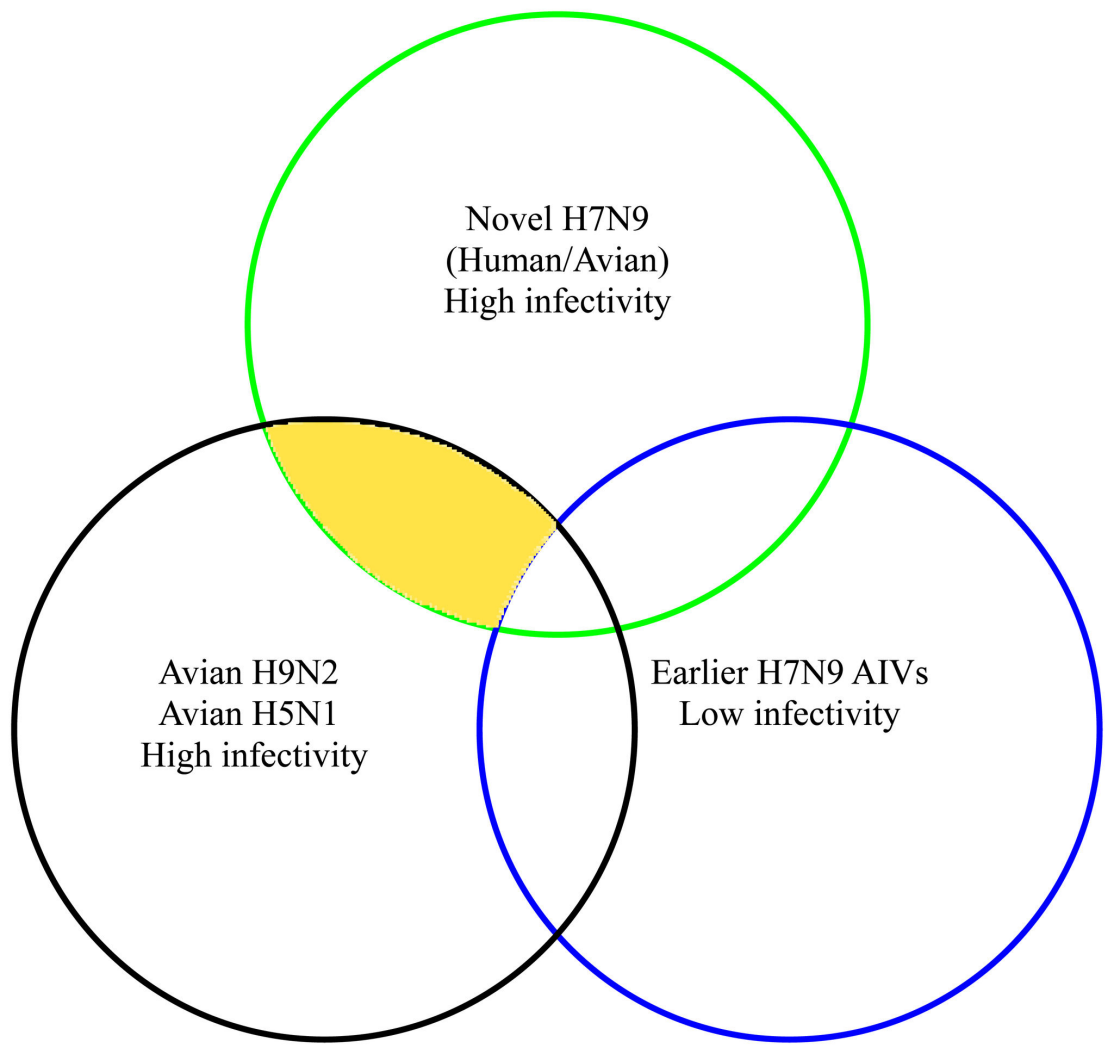
Comparative process on the amino acids associated with high infectivity of the novel H7N9 virus. The green and blue circles represent the novel H7N9 viruses and the earlier H7N9 AIVs respectively. The black circle contains the HPAI H5N1 viruses and the H9N2 AIVs. The infectivity of each group is marked behind the viruses. The yellow regions represent the amino acids associated with high infectivity of the novel H7N9 virus.

In this study, the specific amino acids possibly related to the high infectivity of the novel H7N9 viruses were analyzed based on the criteria illustrated in Figure 3. As the novel H7N9 viruses, the HPAI H5N1 and the H9N2 AIVs were all defined as highly transmissible viruses, the amino acids which were identical amongst these three groups were selected first. Since the earlier H7N9 AIVs were considered as low infectivity, the identical amino acids between the earlier H7N9 viruses and the selected amino acid sites mentioned-above were excluded. The results are shown in Table 3.

**Table 3:** Amino acids associated with high infectivity of the novel H7N9 viruses.

					**PB1**		**NP**				**M1**				**M2**		**NS1**
**Subtype**	**Host**	**Origin**	**Period**	**Strain No.**	**113**		**77**	**105**	**377**		**15**	**101**	**166**		**28**		**207**
H7N9	Avian	China	2013	6	I		R	V	N		I	K	A		V		D
	Human	China	2013	5	I		R	V	N		I	K	A		V		D
H9N2	Avian	Worldwide	2009-2012	22	I		R	V	N		I	K	A		V		D
H5N1	Avian	China	2003-2010	19	I		R	V	N		I	K	A		V		D
H7N9	Avian	Worldwide	2000-2011	9	V		K	M/I	S		V	R	V		I		N

 In accordance with Figure 3 and the results in Table 3, a total of nine amino acids within the internal genes, including one in PB1 (aa113), three in NP (aa77, aa105 and aa377), three in M1 (aa15, aa101 and aa166), one in M2 (aa28), and one in NS1 (aa207) were found to be identical between the novel H7N9, the HPAI H5N1 and the H9N2 AIVs groups, but completely different with the earlier avian H7N9 group. Importantly, no amino acid on the HA and NA genes satisfied this requirement, therefore, only the nine specific amino acids showed a relationship with the high infectivity of the novel H7N9 virus. 

## Discussion

Since March 2013, a severe and fatal respiratory disease, characterized by high fever and severe low respiratory symptoms, has occurred continuously in China, mainly in Zhejiang and Jiangsu province, as well as Shanghai city [1,2,6]. The Chinese national influenza centre (CNIC) identified the pathogen as a triple reassortant avian-origin influenza A (H7N9) virus by testing the clinical specimens of the first three patients; meanwhile, the genetic origins of the viruses were also analyzed [1]. 

The novel H7N9 virus has infected 46 individuals resulting in eight deaths within one month in Zhejiang province, China. Except for the laboratory diagnosis of the cases, the environmental specimens collected from live poultry markets were monitored for H5, H7 and H9 AIVs in order to assess whether the novel H7N9 virus has infected the domestic poultry and circulated in the external environment. Interestingly, 23 specimens were detected to be positive for both H7 and H9 during this surveillance, however, the Ct value of H9 (Ct value 10-14) obtained from real-time RT-PCR assay were significant lower than that of H7 (Ct value 17-35) and all the isolates obtained from the double-positive specimens were identified as H9N2 AIVs, which indicated the co-infection of H7 and H9 has existed in Zhejiang province, and that domestic poultry, especially chicken, has a close relationship with the reassortment between the novel H7N9 and avian H9N2 viruses.

Based on the previous study, A/brambling/Beijing/16/2012 (BJ16), a H9N2 strain isolated from brambling in Beijing in 2012, were identified as the donor of the internal genes of the novel H7N9 virus [1]. However, A/environmental/Zhejiang/16/2013 (H9N2)-like strains showed the higher similarities with the novel H7N9 virus than BJ16. The surveillance on the environmental specimens revealed that A/environmental/Zhejiang/16/2013 (H9N2)-like strains existed and circulated in the domestic poultry in Zhejiang province before the outbreak caused by the novel H7N9 virus. Since most of the human infections with the novel H7N9 virus occurred in the Yangtze River Delta, from an area point of view, it seems more reasonable that the six internal genes of the novel H7N9 virus originated from the viruses reassorted and circulated in the Yangtze River Delta. Unfortunately, there is no information about the avian H9N2 viruses isolated in Jiangsu, Zhejiang province and Shanghai city in 2012, it is therefore difficult to clearly know when the ZJ16 (H9N2)-like viruses emerged, how long they have circulated and who were their original host. 

 Human infection with the HPAI H5N1 virus was first reported in Hong Kong in 1997, resulting in 18 cases with six deaths from May 1997 to February 1998 [31]. The pathogen caused this outbreaks was identified as A/Hong Kong/156/97 (H5N1) strain. Genetic analysis on the whole genome sequence showed that the internal genes of A/Hong Kong/156/97 (H5N1) shared the highest homologies with H9N2 AIVs [31,32]. As same as the A/Hong Kong/156/97 (H5N1) strain, the six internal genes of the novel H7N9 virus were also originated from the H9N2 AIVs. The notable feature of both A/Hong Kong/156/97 (H5N1) and the novel H7N9 viruses is the increase of the infectivity after genetic reassortment, with a large amount of poultry and even human infected. Thus, the internal genes reassorted from H9N2 AIVs may affect the infectivity of the novel H7N9 viruses. 

In this study, several subtypes of AIVs, including HPAI H5N1, avian H9N2 and earlier H7N9 viruses, were included to analyze the possible reason for the high infectivity of the novel H7N9 virus. These three subtypes of AIVs were all of avian origin and have been identified to cause human infections. The differences and similarities of amino acid sequences among the three subtypes of AVIs may result in different infectivity to humans. In this study, the HPAI H5N1, the H9N2 AIVs and the novel H7N9 viruses are characterized as high infectivity, therefore the identical amino acids shared amongst these three groups were selected as the high infectivity related sites, as shown in Figure 3. Since the earlier H7N9 AIVs are acknowledged low transmissibility [29,30], we removed the amino acids that the earlier H7N9 virus possessed from the selected sites. Interestingly, all the nine amino acids obtained, possibly associated with the high infectivity of the novel H7N9 virus, were located on the internal proteins and none were found on the surface proteins. It can be inferred that the internal genes, reassorted from avian H9N2 virus, may increase the infectious ability of the novel H7N9 virus. 

Even though the novel H7N9 virus is highly infective and pathogenic to humans, it is generally low pathogenic to poultry [1,29]. Therefore, it is difficult to eliminate the novel H7N9 virus from the environment, the domestic poultry as well as the wild birds. Although the infection can be controlled in the same manner as the HPAI H5N1 viruses through slaughter of the poultry [31,33,34], widely existing avian H9N2 viruses will provide rich sources of the internal genes to H5, H7 and other subtype viruses [14,19,31]. Once the reassortment happened, the newly reassortant avian influenza viruses have the potential to cross the species barrier to infect humans. Therefore, the surveillance on mutations, evolutionary and reassortment of the H9N2 AIVs should be strengthened for the novel H7N9 viruses to be controlled.

## Materials and Methods

### Ethics Statement

 Ethics committee of Zhejiang provincial Center for Disease Control and Prevention (CDC) approved this study. In order to control the outbreak of infection with the novel H7N9 virus, staff in municipal CDC took responsibility for disinfection of live poultry markets. To assess the disinfectant effect, the environmental specimens were collected before and after the disinfection. All the specimens used in this study were collected from environment before disinfection. 

### Specimens and virus isolation

In total 82 environmental specimens were screened for subtype H5, H7 and H9 AIVs using one-step real-time reverse-transcriptase polymerase chain reaction (rRT-PCR) assays. The primer pairs and probes of the real-time RT-PCR assays were designed and provided by the Chinese national influenza centre (CNIC). The environmental specimens, including swabs of chicken cage, chicken manure and sewage of slaughter poultry, were collected from the live poultry markets in Zhejiang province, China during 1^st^ April to 2^nd^ May in 2013. Those specimens found to be positive for both H7 and H9 AIVs were inoculated to the allantoic sac of 9-to-11-day-old specific pathogen-free (SPF) embryonated chicken eggs for 48 hours at 35°C to propagate the virus. 

### RNA extraction and sequencing

 RNA was extracted from the harvested allantoic liquids using the QIAamp Viral RNA Mini Kit (Qiagen). The whole genomes of five avian H9N2 viruses were performed by second generation sequencing in Applied Biosystems (ABI). The obtained sequences of each gene were aligned in BLAST of NCBI. The complete sequences of five Zhejiang isolates were submitted to GenBank by Zhejiang provincial centre for disease control and prevention (CDC). 

### Reassortment analysis

The entire sequences of a total of 221 influenza viruses were used to perform the recombination analysis. The 221 viruses consisted of (1) five avian H9N2 viruses isolated from environmental specimens in Zhejiang province in 2013, (2) five novel H7N9 viruses, including A/Anhui/1/2013 (AH1), A/Shanghai/1/2013 (SH1), A/Shanghai/2/2013 (SH2), A/Hangzhou/1/2013 (HZ1) and A/Zhejiang/DTID-ZJU01/2013 (ZJU01) [1,2,6,35], (3) 80 of subtype H7 viruses (excluding H7N9), (4) 72 of subtype N9 viruses (except for H7N9), (5) seven of avian H7N9 viruses that were isolated before 2013 as well as (6) 52 of avian H9N2 viruses that were isolated worldwide between 1997 and 2012. The entire sequences of the novel H7N9 viruses were obtained from Global Initiative on Sharing Avian Influenza Data (GISAID) while others were downloaded from GenBank of NCBI. 

A multiple sequence alignment was performed by the ClustalW program and the amino acid mutations were analyzed with the Highlight model using MEGA software (Version 5.0). Phylogenetic trees were drawn with the Neighbor-joining method of the MEGA software (Version 5.0) by means of bootstrap analysis with 1000 replications. Reassortment events on the six internal genes of the novel H7N9 viruses were searched by the RDP, GENECONV and MaxChi suites within the Recombination Detection Program (RDP3) software, according to the instruction and previous studies [36]. The highest P value was set as 0.01 in order to enhance the credibility of the results. Other parameters were used with default settings.

### Phylogeny analysis

 Phylogenetic trees were generated based on the amino acid sequences of 39 influenza viruses by means of the Neighbor-joining (NJ) method of the MEGA software (Version 5.0), with the bootstrap analysis of 1000 replications. The 39 viruses contained 19 of avian H9N2 viruses isolated during 1997 to 2012, five HPAI H5N1 viruses isolated in Hong Kong and mainland China, four avian H7N9 viruses isolated worldwide during 2006 to 2011, five novel H7N9 viruses isolated in 2013, five avian H9N2 virus isolated from Zhejiang province in 2013, as well as one avian H7N9 virus A/chicken/Zhejiang/DTID-ZJU01/2013, which was isolated from chicken in Zhejiang province in 2013

### Comparison on the amino acid sequences of three subtypes of AIVs

 The multiple sequence alignments based on the amino acid sequences of the HPAI H5N1 viruses, the H9N2 and the earlier H7N9 AIVs, as well as the novel H7N9 viruses were performed by using BioEdit software. Except for the novel H7N9 viruses, all the amino acid sequences were downloaded from GenBank. The GenBank accession numbers of the AIVs used for the comparison were shown in Table S1.

We classified all the AIVs used in this comparison into four groups based on their different infectivity described in the previous studies [1,2,9–13,19,21–30]. The novel H7N9 group, including five strains isolated from human and six strains isolated from domestic poultry during the outbreaks happened in China in 2013, was defined as highly infective viruses since the novel H7N9 viruses have caused infections of a large amount of domestic poultry and humans in a short time [1-3]. The HPAI H5N1 group, containing 19 strains isolated from poultry in mainland China during 2003 and 2010, as well as the avian H9N2 group, consisting of 22 strains isolated from poultry between 2009 and 2012 worldwide, were also defined as highly transmissible viruses according to the host range, numbers of cases and seropositive rates in both poultry and human [10-13,21-28]. However, the earlier avian H7N9 virus, comprising nine strains isolated from avian species worldwide between 2000 and 2011, was defined as low infective viruses. 

The comparative method and selection principle of the specific amino acids associated with high infectivity of the novel H7N9 virus is illustrated in Figure 3. The amino acids sequences on the whole genome of the four group AIVs were compared. The yellow region, representing high infectivity, was obtained by selecting the amino acids that were identical amongst the novel H7N9, the HPAI H5N1 and H9N2 AIVs, but different from the earlier H7N9 AIVs. 

## Supporting Information

Table S1 **Genbank accession numbers of amino acid sequences for the AIVs used in this study.**
(DOC)
