# Supplementary material for: Origin and Characteristics of Internal Genes Affect Infectivity of the Novel Avian-Origin Influenza A (H7N9) Virus
Source: PLoS One. 2013 Nov 22;8(11):e81136. doi: 10.1371/journal.pone.0081136 (PMC3838381; doi:10.1371/journal.pone.0081136)
Supplement: Table S1 — Genbank accession numbers of amino acid sequences for the AIVs used in this study. (DOC) [file pone.0081136.s001.doc]

**Table S1 GenBank accession numbers of amino acid sequences for the AIVs used in this study**

| **Subtype** | | **Host** | | **Strain name** | **Accession Numbers** | | | | | |
| --- | --- | --- | --- | --- | --- | --- | --- | --- | --- | --- |
|  |  |  |  |  | **PB1** | | **NP** | **M1** | **M2** | **NS1** |
| H7N9 | | Avian | | A/chicken/Zhejiang/DTID-ZJU01/2013 | AGJ72859 | | AGJ72862 | AGJ72864 | AGJ72865 | AGJ72866 |
|  |  |  |  | A/chicken/Guangdong/SD641/2013 | AGR49335 | | AGR49340 | AGR49342 | AGR49343 | AGR49344 |
|  |  |  |  | A/chicken/Jiangsu/S002/2013 | AGR49347 | | AGR49352 | AGR49354 | AGR49355 | AGR49356 |
|  |  |  |  | A/chicken/Shanghai/017/2013 | AGU70024 | | AGU70019 | AGU70016 | AGU70017 | AGU70020 |
|  |  |  |  | A/chicken/Zhejiang/SD007/2013 | AGR49526 | | AGR49531 | AGR49533 | AGR49534 | AGR49535 |
|  |  |  |  | A/duck/Anhui/SC702/2013 | AGR49562 | | AGR49567 | AGR49569 | AGR49570 | AGR49571 |
|  |  | Human | | A/Zhejiang/DTID-ZJU01/2013 | AGJ51960 | | AGJ51954 | AGJ51957 | AGJ51956 | AGJ51959 |
|  |  |  |  | A/Hangzhou/1/2013 | AGK84851 | | AGK84853 | AGI60298 | AGI60299 | AGK84855 |
|  |  |  |  | A/Shanghai/02/2013 | AGL44434 | | AGL44439 | AGL44441 | AGL44442 | AGL44443 |
|  |  |  |  | A/Wuxi/1/2013 | AGN69470 | | AGN69475 | AGN69477 | AGN69478 | AGN69479 |
|  |  |  |  | A/Zhejiang/HZ1/2013 | AGM16239 | | AGM16236 | AGM16243 | AGM16244 | AGM16240 |
| H9N2 | | Avian | | A/chicken/Shandong/01/2009 | AEB71301 | | AEB71259 | AEB71231 | AEB71232 | AEB71201 |
|  |  |  |  | A/chicken/Tibet/S4/2009 | ADZ45214 | | ADZ45218 | ADZ45220 | ADZ45221 | ADZ45222 |
|  |  |  |  | A/chicken/Tibet/S1/2009 | ADZ45203 | | ADZ45207 | ADZ45209 | ADZ45210 | ADZ45211 |
|  |  |  |  | A/duck/Tibet/S2/2009 | ADZ45225 | | ADZ45229 | ADZ45231 | ADZ45232 | ADZ45233 |
|  |  |  |  | A/duck/Shanghai/C163/2009 | AGI55558 | | AGI55575 | AGI55582 | AGI55583 | AGI55570 |
|  |  |  |  | A/duck/Shanghai/C164/2009 | AGI55560 | | AGI55576 | AGI55584 | AGI55585 | AGI55572 |
|  |  |  |  | A/chicken/Jiangsu/Q3/2010 | AET10134 | | AET10132 | AET10130 | AET10129 | AET10128 |
|  |  |  |  | A/chicken/Shandong/03/2010 | AEB71291 | | AEB71249 | AEB71211 | AEB71212 | AEB71181 |
|  |  |  |  | A/chicken/Shandong/BD/2010 | AEB71293 | | AEB71251 | AEB71215 | AEB71216 | AEB71185 |
|  |  |  |  | A/chicken/China/AH-10-01/2010 | AEE73581 | | AEE73585 | AEE73587 | AEE73588 | AEE73589 |
|  |  |  |  | A/chicken/Zhejiang/329/2011 | AEY75684 | | AEY75681 | AEY75686 | AEY75687 | AEY75688 |
|  |  |  |  | A/chicken/Guangdong/ZHJ/2011 | AET10172 | | AET10170 | AET10168 | AET10167 | AET10166 |
|  |  |  |  | A/chicken/Zhejiang/611/2011 | AEY75662 | | AEY75659 | AEY75664 | AEY75665 | AEY75666 |
|  |  |  |  | A/chicken/Zhejiang/607/2011 | AEY75673 | | AEY75670 | AEY75675 | AEY75676 | AEY75677 |
|  |  |  |  | A/chicken/Guangdong/ZCY/2011 | AET10162 | | AET10160 | AET10158 | AET10157 | AET10156 |
|  |  |  |  | A/chicken/Shanghai/C1/2012 | AGC74222 | | AGC74237 | AGC74243 | AGC74244 | AGC74231 |
|  |  |  |  | A/chicken/Shanghai/C2/2012 | AGC74224 | | AGC74238 | AGC74245 | AGC74246 | AGC74233 |
|  |  |  |  | A/chicken/Shanghai/C3/2012 | AGC74226 | | AGC74239 | AGC74247 | AGC74248 | AGC74235 |
|  |  |  |  | A/brambling/Beijing/16/2012 | AGE15506 | | AGE15509 | AGE15512 | AGE15511 | AGE15514 |
|  |  |  |  | A/Japanese Quail/Vietnam/4/2009 | BAN15776 | | BAN15779 | BAN15782 | BAN15781 | BAN15784 |
|  |  |  |  | A/Rock Pigeon/Vietnam/6/2009 | BAN15787 | | BAN15790 | BAN15793 | BAN15792 | BAN15795 |
|  |  |  |  | A/African Stonechat/Vietnam/8/2009 | BAN15808 | | BAN15812 | BAN15817 | BAN15816 | BAN15815 |
| H5N1 | | Avian | | A/duck/China/E319-2/03 | AAR99632 | | AAR99630 | AAR99627 | AAR99626 | AAR99625 |
|  |  |  |  | A/wild duck/Hunan/021/2005 | ABX83936 | | ABX83939 | ABX83941 | ABX83942 | ABX83943 |
|  |  |  |  | A/chicken/Hebei/A-8/2009 | ADG59438 | | ADG59241 | ADG59133 | ADG59134 | ADG59305 |
|  |  |  |  | A/duck/Hubei/wp/2003 | ABJ09482 | | ABJ09478 | ABJ09484 | ABJ09485 | ABJ09479 |
|  |  |  |  | A/chicken/Henan/12/2004 | AAX53560 | | AAX53533 | AAX53515 | AAX53516 | AAX53543 |
|  |  |  |  | A/duck/Guangdong/173/04 | AAW59406 | | AAW59409 | AAW59410 | AAW59411 | AAW59412 |
| H5N1 | Avian | | A/chicken/Xinjiang/53/2005 | | ADG59467 | ADG59275 | | ADG59135 | ADG59136 | ADG59365 |
|  |  |  | A/chicken/Xinjiang/53/2005 | | ADG59467 | ADG59275 | | ADG59135 | ADG59136 | ADG59365 |
|  |  |  | A/duck/Anhui/56/2005 | | ADG59474 | ADG59251 | | ADG59163 | ADG59164 | ADG59303 |
|  |  |  | A/chicken/Sichuan/81/2005 | | ADG59429 | ADG59238 | | ADG59095 | ADG59096 | ADG59285 |
|  |  |  | A/duck/Jiangxi/80/2005 | | ADG59463 | ADG59266 | | ADG59161 | ADG59162 | ADG59343 |
|  |  |  | A/chicken/Shanxi/2/2006 | | ABK34761 | ABK34765 | | ABK34768 | ABK34767 | ABK34770 |
|  |  |  | A/duck/Yunnan/5310/2006 | | ACH85385 | ACH85389 | | ACH85391 | [ACH85392](http://www.ncbi.nlm.nih.gov/entrez/viewer.fcgi?val=ACH85392) | [ACH85393](http://www.ncbi.nlm.nih.gov/entrez/viewer.fcgi?val=ACH85393) |
|  |  |  | A/duck/Hubei/Hangmei01/2006 | | ACF16398 | ACF16401 | | ACF16403 | ACF16404 | ACF16405 |
|  |  |  | A/chicken/Fujian/1/2007 | | ADG59445 | ADG59247 | | ADG59103 | ADG59104 | ADG59307 |
|  |  |  | A/duck/Hunan/3/2007 | | ACZ05919 | ACZ05877 | | ACZ05847 | ACZ05848 | ACZ05819 |
|  |  |  | A/chicken/Huadong/4/2008 | | AFR53947 | AFR53949 | | AFR53950 | AFR53951 | AFR53952 |
|  |  |  | A/chicken/Shandong/A-1/2009 | | ADG59435 | ADG59276 | | ADG59165 | ADG59166 | ADG59329 |
|  |  |  | A/chicken/Jiangsu/k0402/2010 | | AFC98314 | AFC98306 | | AFC98300 | AFC98301 | AFC98308 |
| H7N9 | Avian | | A/ruddy turnstone/Delaware/AI00-1538/2000 | | AGL57446 | AGL57439 | | AGL57436 | AGL57437 | AGL57440 |
|  |  |  | A/duck/Jiangxi/3096/2009 | | AGQ83196 | AGQ81982 | | AGQ81219 | AGQ81220 | AGQ82226 |
|  |  |  | A/duck/Jiangxi/3214/2009 | | AGQ83204 | AGQ81986 | | AGQ81227 | AGQ81228 | AGQ82234 |
|  |  |  | A/duck/Jiangxi/3257/2009 | | AGQ83306 | AGQ82037 | | AGQ81329 | AGQ81330 | AGQ82336 |
|  |  |  | A/American green-winged teal/Mississippi/11OS259/2011 | | AGE08179 | AGE08175 | | AGE08170 | AGE08171 | AGE08176 |
|  |  |  | A/blue-winged teal/Ohio/566/2006 | | ABS89417 | ABS89413 | | ABS89410 | ABS89411 | ABS89414 |
|  |  |  | A/Anas crecca/Spain/1460/2008 | | ADN34735 | ADN34739 | | ADN34741 | ADN34742 | ADN34743 |
|  |  |  | A/blue-winged teal/Guatemala/CIP049-01/2008 | | ADK71134 | ADK71138 | | ADK71140 | ADK71141 | ADK71142 |
|  |  |  | A/northern shoverl/Mississippi/11OS145/2011 | | AGE08106 | AGE08102 | | AGE08099 | AGE08100 | AGE08103 |
